# Deep Learning-Based CT-Less Cardiac Segmentation of PET Images: A Robust Methodology for Multi-Tracer Nuclear Cardiovascular Imaging

**DOI:** 10.1007/s10278-025-01528-0

**Published:** 2025-05-06

**Authors:** Yazdan Salimi, Zahra Mansouri, René Nkoulou, Ismini Mainta, Habib Zaidi

**Affiliations:** 1 Division of Nuclear Medicine and Molecular Imaging, Geneva University Hospital, CH-1211 Geneva, Switzerland; 2 Department of Nuclear Medicine and Molecular Imaging, University Medical Center Groningen, University of Groningen, Groningen, Netherlands; 3 Department of Nuclear Medicine, University of Southern Denmark, Odense, Denmark; 4 University Research and Innovation Center, Óbuda University, Budapest, Hungary

**Keywords:** PET/CT, Cardiovascular imaging, Deep learning, Segmentation, Quantification

## Abstract

Quantitative cardiovascular PET/CT imaging is useful in the diagnosis of multiple cardiac perfusion and motion pathologies. The common approach for cardiac segmentation consists in using co-registered CT images, exploiting publicly available deep learning (DL)-based segmentation models. However, the mismatch between structural CT images and PET uptake limits the usefulness of these approaches. Besides, the performance of DL models is not consistent over low-dose or ultra-low-dose CT images commonly used in clinical PET/CT imaging. In this work, we developed a DL-based methodology to tackle this issue by segmenting directly cardiac PET images. This study included 406 cardiac PET images from 146 patients (43 ^18^F-FDG, 329 ^13^N-NH_3_, and 37 ^82^Rb images). Using previously trained DL nnU-Net models in our group, we segmented the whole heart and the three main cardiac components, namely the left myocardium (LM), left ventricle cavity (LV), and right ventricle (RV) on co-registered CT images. The segmentation was resampled to PET resolution and edited through a combination of automated image processing and manual correction. The corrected segmentation masks and SUV PET images were fed to a nnU-Net V2 pipeline to be trained in fivefold data split strategy by defining two tasks: task #1 for whole cardiac segmentation and task #2 for segmentation of three cardiac components. Fifteen cardiac images were used as external validation set. The DL delineated masks were compared with standard of reference masks using Dice coefficient, Jaccard distance, mean surface distance, and segment volume relative error (%). Task #1 average Dice coefficient in internal validation fivefold was 0.932 ± 0.033. The average Dice on the 15 external cases were comparable with the fivefold Dice reaching an average of 0.941 ± 0.018. Task #2 average Dice in fivefold validation was 0.88 ± 0.063, 0.828 ± 0.091, and 0.876 ± 0.062 for LM, LV, and RV, respectively. There was no statistically significant difference among the Dice coefficients, neither between images acquired by three radiotracers nor between the different folds (*P*-values >  > 0.05). The overall average volume prediction error in cardiac components segmentation was less than 2%. We developed an automated DL-based segmentation pipeline to segment the whole heart and cardiac components with acceptable accuracy and robust performance in the external test set and over three radiotracers used in nuclear cardiovascular imaging. The proposed methodology can overcome unreliable segmentations performed on CT images.

**Supplementary Information:**

The online version contains supplementary material available at 10.1007/s10278-025-01528-0.

## Introduction

Cardiovascular PET/CT imaging is used in a number of clinical indications, including the assessment of viability, perfusion, and motion abnormalities. Cardiac PET image quantification is useful in both clinical diagnosis and prognosis of patients undergoing ^18^F-sodium fluoride (^18^F-NaF) cardiac PET examinations through calculating factors, such as Alavi-Carlsen calcification score [1–4]. However, PET quantification requires reliable segmentation, which remains the bottleneck since it is time consuming and requires expert guidance [3]. The first step in cardiac PET quantification is to delineate different areas. The segmented images can be used for evaluation of the extent and severity of any cardiac pathology, such as stress-induced ischemia or motion problems which is not limited to the left ventricle. Radiomic studies using machine learning have shown good capability in both clinical diagnosis and prognosis/outcome prediction [5, 6]. However, image segmentation is a requirement to carry out radiomics analysis [7, 8]. Deep learning (DL)–based segmentation algorithms has shown promising results in the delineation of multiple organs and cardiac components on CT images [9, 10]. The hypothesis is that PET and CT images acquired in a single session are inherently co-registered, and as such, the segmentation generated on CT images can be transferred to PET images after resampling. Yet, the high prevalence of mismatch between PET and CT images either due to voluntary bulk motion or involuntary cardiac and respiratory motion challenges this strategy [11]. This fact limits the application and usefulness of CT segmentation approaches in cardiac quantification especially for dynamic imaging procedures where the CT acquired at the beginning or at the end of the scan may be different than the rest of dynamic PET images. On the other hand, gated cardiac PET images are common for evaluation of motion and thickening of myocardial wall and ejection fraction (EF) calculation. The CT-based generated masks match only with one of the 8 or 16 gating phases in the best-case scenario. Besides, the performance of DL-based cardiac cavities segmentation on unenhanced low-dose CT images acquired for attenuation and scatter correction (CTAC) may be questionable especially when a trained human eye cannot easily distinguish the LV myocardium from LV cavity on CT images. Ultra-low-dose CTAC images suffer from artifacts due to photo deprivation and beam hardening, limiting the performance of available DL-based segmentation methods trained on high quality and contrast-enhanced CT images. The CT radiation dose can be reduced further using special beam filtration, which produces noisier images, yet sufficient for PET quantification [12, 13]. In addition, recent developments in CT-less PET and SPECT imaging using deep learning algorithms [14–17] emphasizes the need for a robust automated segmentation tool using only the emission data owing to the lack of the CT component. In addition, heart motion due to respiration and heart beating results in movement, contraction, and changes in the heart cavities and muscles shape, which cannot be accurately corrected using manual registration of PET and CT images.

According to our clinical experience, the DL segmentation output on noisy CT images may visually seem very promising as it reproduces the same shape as in the training dataset with the borders inside the heart tissue. However, the CT segmented cardiac subsegments are not necessarily aligned with the PET uptake [18]. This could be due to either a misalignment between PET and CT or an error in the DL-based CT segmentation. In the first step, we explored the two mentioned reasons of mismatch between CT segmentation and PET uptake by testing two available cardiac DL-based segmentation tools [9, 10] on few samples from our local data.

Using emission data for DL-based organ segmentation has been previously reported. Entezarmahdi et al. [19] developed a semi-automated segmentation method to segment the right ventricle (RV) on short-axis reoriented SPECT images to evaluate the RV quantitatively on non-gated SPECT images. Sadhanandan et al. [20] developed a DL model to segment cardiac components on cardiac MR images, emphasizing the importance of robust segmentation on gated images. Szűcs et al. [21] developed a model to segment the left ventricle (LV) on reoriented short-axis SPECT cardiac images, highlighting the robustness of their model in cases with severe stress-induced cardiac ischemia. Yazdani et al. [22] developed DL segmentation models to segment both healthy organs and tumoral tissues from ^68^ Ga-PSMA PET/CT images. Wang et al. [23] segmented bladder and heart organs on ^18^F-FDG PET/CT images to overcome the limitations related to the lack or availability of unreliable CT images. Zhang et al. [24] segmented left myocardium (LM) and LV cavity on dynamic cardiac ^13^N-NH_3_ reoriented short-axis PET/CT images. They generated manual segmentation on PET images and used their model to extract the radiomics information from cardiac images cropped and reoriented manually to short-axis direction. Salimi et al. [25] developed a multiple total body organ segmentation pipeline based on PET emission images to be used when CT-based image segmentations are unreliable owing to misregistration. Studies have shown the importance of right ventricle volume RV volume in diagnosis and prognosis of cardiac diseases [26]. Josselyn [27] developed a segmentation mode for left ventricle delineation on PET images and reported its importance in extracting cardiac uptake patterns. The goal of this study was to tackle the challenges of unreliable CT segmentation by developing robust DL algorithms to segment the whole heart and its cardiac components using only the PET emission data from multiple tracers commonly used for cardiac imaging.

## Materials and Methods

### Understanding Underperformance

To understand the reasons of the misalignment between PET tracer uptake and CT segmentation masks, we tested two publicly available DL-based CT cardiac segmentation pipelines from Wasserthal et al. (model #1) [9] and Salimi et al. (model #2) [10], both trained using nnU-Net on a group of low-dose CTAC and standard-dose CT images with and without contrast enhancement, respectively. We generated segmentation masks using different types of CT images as input to these models and compared the segmentation outputs with PET cardiac uptake. We included different samples as test dataset in this step, including sample (#1) a high-resolution contrast-enhanced cardiac CT image acquired on a dual-energy CT scanner, sample (#2) three unenhanced cardiac PET/CT images acquired with low-dose CTAC protocol, sample (#3) three unenhanced ultra-low-dose cardiac CT images, and sample (#4) A PET/CT image with two co-registered CT images, one unenhanced low-dose CTAC, and one contrast-enhanced venous phase standard-dose CT. The last image was used to compare two models’ performance on high-quality and low-dose CT images of the same patient. In addition, one could argue that the DL network detects contrast information not perceptible by the human eye. To control this, we used sample #4 images and removed all available contrast media inside the heart. To explain and understand the behavior of deep learning algorithms, usually referred to as a black box, we segmented the whole heart from CT images, then replaced the values inside the heart segment with a random image from − 100 to 100 HU and inferenced the DL segmentation on the updated images. The bottom part of Fig. 2 shows an example of a CT image with a heart region replaced by a random image. First, a random image with the same size as the CT image was generated using NumPy Python library. Then, all voxels were replaced by original CT values, except voxels where the whole heart segmentation value was not zero.

The previous step was carried out to explain the nnU-NET CT segmentation model in cases where the human expert eye could not easily distinguish between the myocardium and cardiac cavities. The evaluations showed that using unenhanced low-dose CT images is not a reliable approach for cardiac components, reinforcing the need for developing a reliable segmentation tool using cardiac PET images. Based on the results achieved in this step, we attempted to train PET-only segmentation models to tackle this issue. All images were collected at Geneva University Hospital (HUG), Geneva, Switzerland. The images were acquired on three different Siemens PET/CT scanners.

### PET-Only Segmentation Option

This step included 406 cardiac PET images from 146 patients (42 with ^18^F-FDG, 329 with ^13^N-NH_3_, and 37 with ^82^Rb). Patient demographics, acquisition, and reconstruction parameters are summarized in Table 1. CT and PET cardiac images were converted to NIfTI format and then PET images were converted to standardized uptake value (SUV) normalized to body weight using Eq. 1 and the information stored in the DICOM header. It should be mentioned that only PET attenuation and scatter corrected images were included in this study. For training purpose, the stress and rest summed image, i.e., summation of 8 gated phases, was used. However, for the testing dataset, both summed and single-phase gated images were used.

**Table 1:** Summary of patient demographics and CT and PET image acquisition and reconstruction parameters

	Procedure–subtype	^18^F-FDG	^13^N-NH_3_	^82^Rb
Patient	# of studies	20	120	6
	# of images	42	328	36
	Age (years)	68.8 ± 9.2 (54–90)	51.9 ± 16.7 (22–85)	60.0 ± 11.6 (43–75)
	Gender	M, 18; F, 2	M, 81; F, 39	F, 3; M, 3
	Study date	03/12/2008–31/01/2023	07/11/2008–25/04/2018	07/07/2021–22/05/2023
	Weight (Kg)	81.0 ± 13.3 (45.0–105.0)	85.9 ± 23.2 (42.0–146.0)	70.4 ± 8.4 (59.0–84.0)
CT	Pixel spacing (mm)	1.37 ± 0.03 (1.36–1.52)	1.36 ± 0.03 (0.97–1.36)	1.52
	kVp	120	120	100, 120, 140
	Manufacturer	Siemens	Siemens	Siemens
	Slice thickness (mm)	2.95 ± 0.21 (- to 3)	2.99 ± 0.09 (2–3)	3
	Kernel	B18 s, Bf37f	B18 s, B20f	I30f
	Average tube current (mA)	38.5 ± 19.7 (34.0–124.5)	34.1 ± 1.4 (31.1–50.0)	120.4 ± 19.0 (98.3–148.8)
PET	Model	Biograph True Point, Biograph Vision 600	Biograph True Point	Biograph mCT
	Pixel spacing (mm)	2.35 ± 0.55 (1.33–2.67)	2.45 ± 0.55 (1.33–4.07)	2.03
	Slice thickness (mm)	2.01 ± 0.08 (1.64–2.02)	2	3
	Injected activity (MBq)	395.8 ± 52.5 (223–461)	567.2 ± 14.9 (259–1096)	600
	Scatter correction method	Model-based	Model-based	Model-based
	Reconstruction method	OSEM3D 6i8 s, PSF + TOF 5i5 s	OSEM3D 6i8 s, OSEM3D 4i8 s	OSEM3D PSF + TOF 3i21 s

1 $$SUV=\frac{Activity\;concentration(\frac{Bq}{ml})}{Injected\;Activity\;\left(Bq\right)/Patient\;Weight\;(g)}$$

Whole heart and heart components, including the LM, LV, and RV, were delineated on CT images using previously trained nnU-Net models in our lab [10]. The segmentations were resampled to PET resolution and visualized on PET/CT images using commercial ITK-SNAP software [28] to ensure acceptable match between the segmentation masks, CT, and PET images. As expected, after visual assessment of the first 50 images, only 8 cases presented with good co-registration accuracy between the CT LV myocardium and the corresponding PET uptake, thus necessitating a correction of the segmentation output before training the PET segmentation model. To accomplish this, we edited the segmentation masks generated on CT images as described in the next step.

The segmentation masks were corrected using a combination of thresholding on PET images (empirical threshold of 2 SUV) and dilation of the CT segmentation mask with multiple conditions and image processing steps considering the best accuracy in LM. First, the PET upper than threshold segmentations (PET-LM-threshold) were saved. Then, the CT LM segmentations were dilated by 2 mm and combined with PET thresholding-based segmentation. The segmented PET image contained voxels outside the heart volume. The voxels outside the whole heart segmented on the CT image were removed.

**Fig. 1:**
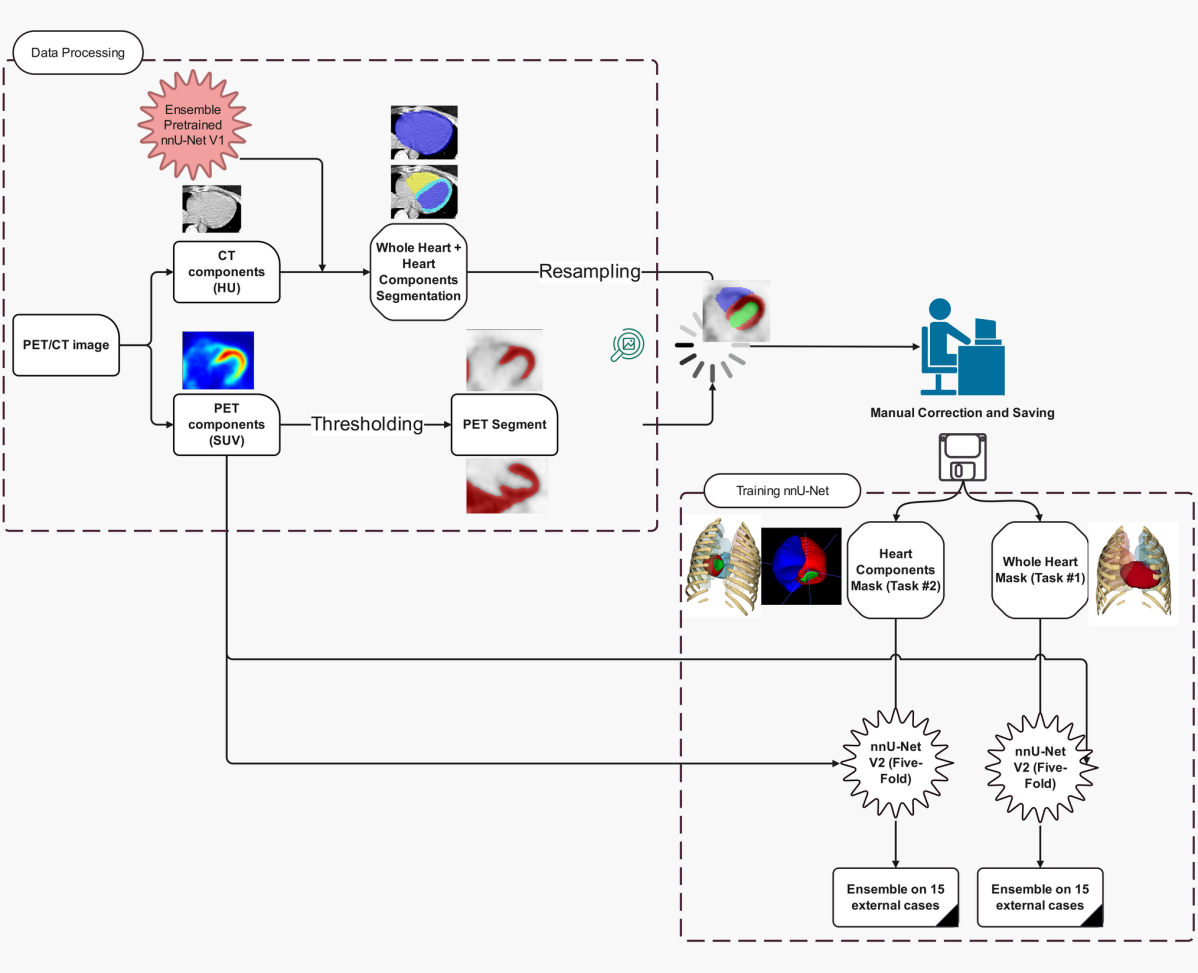
Flowchart adopted to generate reference segmentations and the data flow adopted in our study. The ribs and lungs segmentations are shown for better visualization

Figure 1 shows an example of the steps followed to generate PET matched segmentations. The LV and RV segmentations were copied to the new segmentation mask. The codes explaining the different steps are presented in the supplementary material. All images and heart components segmentation pairs were visually checked, and segmentations manually edited if necessary. Two separate tasks were defined, task #1, segmentation of whole cardiac tissue as a single mask covering all right and left ventricles and atriums; and task #2, segmentation of selected cardiac components including LM, LV, and RV. Two separate models were trained in a fivefold data split scheme.

PET and refined segmentation pairs were fed to an nnU-Net model [29] version 2 (nnunetv2) pipeline in a fivefold data split strategy making sure that multiple images from the same study (stress and rest or repeated scans) are all in the train or test split, i.e., both stress and rest images of patient #1 were in the test or both in training set for each fold to prevent overfitting and any kind of data leakage. The nnU-Net configuration was updated to 2000 epochs training duration, and the rest of parameters were kept as default, i.e., initial learning rate of 1e-2, decay 3e-5, Dice-cross entropy loss function, 250 iterations per epoch during training, and 8 GB GPU memory target. The training was carried out on a PC equipped with RTX 4090 GPU with 24 GB of dedicated memory and Corei913900 KF CPU with 32 GB of memory. The same nnU-Net configuration was used to train separate pipelines for task #1 and task #2.

The fivefold models were saved and tested on 15 external cases, including 5 cases from each radiopharmaceutical (42 ^18^F-FDG, 329 ^13^N-NH_3_, and 37 ^82^Rb) through ensembling all folds on the images as described in nnU-Net inference methods [29]. The internal fivefold validation using single model and external validation using five models’ outputs were compared with reference segmentation masks in terms of Dice coefficient, Jaccard, and mean surface distance. In addition, the segment volume of all heart components was calculated on reference and predicted images and the volume relative error (volume-RE%) calculated according to Eq. 2.

2 $$volume-RE\%=\frac{predicted\;volume-reference\;volume}{reference}volume\times100\%$$

To check the robustness of our models, we included few examples of gated cardiac PET images acquired using Siemens CardioFreeze software (Siemens Healthineers, Knoxville, USA) and few dynamic cases where the dynamic frames were acquired after injection of the radiotracer. Mann–Whitney test was used to compare model performance among the different tracers using SciPy version 1.11.2 python library. *P*-values higher than 0.05 were considered statistically significant. We compared multiple image segmentation metrics including Dice coefficient, Jaccard, mean surface distance, volume difference, and volume-RE% in three segmentation masks including the left myocardium, left ventricle cavity, and right ventricle. First, we used Kolmogorov Smirnov statistical test for testing the normality of data distributions, and the results showed that none of the metric distributions is normal with *P*-values lower than 0.05. To do so, we used Kruskal–Wallis non-parametric test to compare three groups together and in cases of significant difference among them, at the next step, we completed the evaluation using post-hoc Dunn test to compare them two by two and understand the differences.

## Results

### Understanding Underperformance

**Fig. 2:**
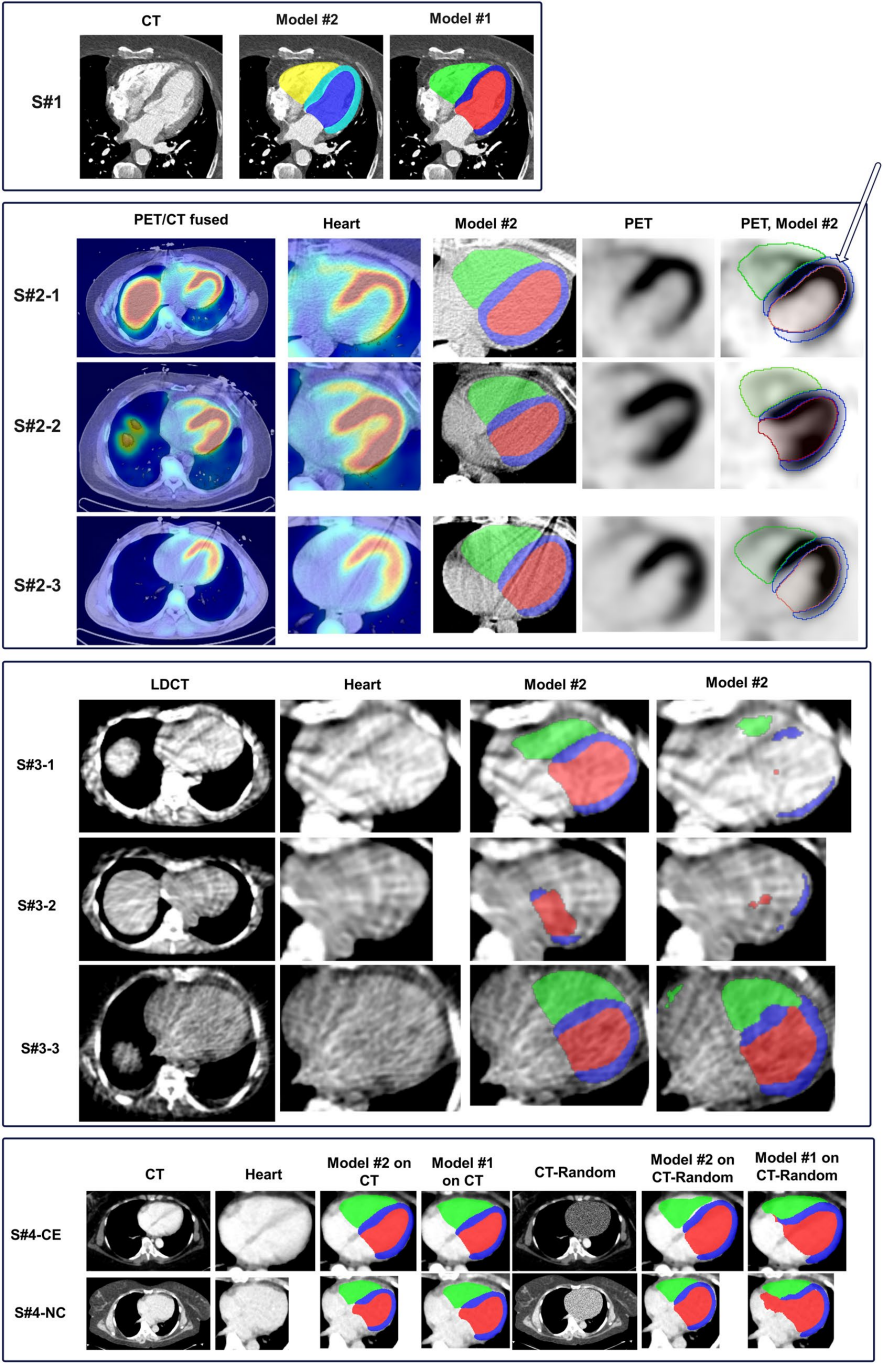
DL CT inferences. From top to bottom sample #1 (S#1) to sample #4 (S#4). S#4–1 windowing (− 110 to 170). S#4–2 windowing (− 60 to 160 HU)

Figure 2 shows an example of low-dose and ultra-low-dose CT images along with segmentations generated using them as input to deep learning models. The models’ performance was excellent on a contrast-enhanced standard dose CT of sample #1, while model #1 [9] offered a finer segmentation. On sample #2, the CT-generated segmentation by model #2 matched well one of the three cases (middle case in Fig. 2, S#2–2), while there was unacceptable mismatch between them on the other two samples (S#2–1 and S#2–3). Sample #3 consisted of ultra-low-dose CTAC images including three images, namely model #1 comprising generated cardiac components only on one of them and model #2 on two of them. Even though, the contours were not discernible on the heart by the human eye. Finally, on sample #4 where we replaced the intensities inside the whole heart with random values, model #1 and model #2 reproduced the segmentation masks with almost the same shape as the usual cardiac components’ shape, while the input to these models were only random images inside the cardiac region.

### PET‑Only Segmentation Solution

Task #1’s average Dice in the fivefold internal validation set was 0.932 ± 0.033. The detailed results of the fivefold validation for task #1 are shown in Table 2. The average Dice on the 15 external cases was comparable with the fivefold Dice, with an average value and SD of 0.941 ± 0.018. Task #2’s average Dice in fivefold validation was 0.88 ± 0.063, 0.828 ± 0.091, and 0.876 ± 0.062 for LM, LV, and RV, respectively. There was no statistically significant difference neither between the Dice values achieved for images acquired using the three different radiotracers, nor between the different folds (*P*-values > 0.05). The detailed performance of fivefold validation metrics separated by folds is presented in Table 3. The overall average volume prediction error in LV cavity segmentation was less than 2%.

**Table 2:** Task #1 fivefold validation metrics

Fold–subtype	Dice	Jaccard	Sensitivity	Mean surface distance
fold_0	0.933 ± 0.036 (0.81–0.972)	0.876 ± 0.059 (0.681–0.946)	0.932 ± 0.043 (0.778–0.987)	1.898 ± 1.118 (0.626–5.578)
fold_1	0.927 ± 0.036 (0.777–0.976)	0.865 ± 0.06 (0.635–0.953)	0.926 ± 0.045 (0.776–0.994)	2.151 ± 1.212 (0.466–7.119)
fold_2	0.931 ± 0.034 (0.848–0.976)	0.873 ± 0.057 (0.736–0.953)	0.942 ± 0.04 (0.837–0.989)	2.053 ± 1.278 (0.553–5.757)
fold_3	0.938 ± 0.025 (0.871–0.973)	0.884 ± 0.043 (0.771–0.948)	0.94 ± 0.03 (0.825–0.985)	1.823 ± 0.938 (0.555–4.771)
fold_4	0.933 ± 0.033 (0.813–0.977)	0.876 ± 0.055 (0.685–0.956)	0.939 ± 0.037 (0.825–0.986)	2.007 ± 1.254 (0.511–7.034)
Overall	0.932 ± 0.033 (0.777–0.977)	0.875 ± 0.056 (0.635–0.956)	0.936 ± 0.04 (0.776–0.994)	1.986 ± 1.172 (0.466–7.119)

**Table 3:** Task #2 performance metrics for internal validation evaluation

Fold–subtype	Segment–subtype	Dice	Jaccard	Mean surface distance	Volume difference	Actual volume	Predicted volume	Volume-RE%
fold_0	LV Cavity	0.834 ± 0.087 (0.579–0.964)	0.724 ± 0.121 (0.408–0.93)	1.411 ± 0.912 (0.2–3.905)	− 0.895 ± 3.355 (− 10.586–7.334)	50.255 ± 16.946 (23.091–90.675)	49.36 ± 15.715 (24.098–92.339)	− 0.769 ± 6.883 (− 14.642–19.865)
fold_0	LV Myocardium	0.878 ± 0.068 (0.619–0.968)	0.789 ± 0.101 (0.448–0.939)	1.48 ± 1.039 (0.239–5.646)	3.327 ± 12.735 (− 19.211–35.298)	284.222 ± 53.745 (189.769–420.883)	287.549 ± 53.451 (195.816–420.999)	1.353 ± 4.964 (− 6.356–16.603)
fold_0	RV	0.876 ± 0.065 (0.696–0.958)	0.785 ± 0.097 (0.534–0.919)	1.797 ± 1.888 (0.413–13.942)	− 1.408 ± 14.015 (− 42.994–28.857)	212.353 ± 47.883 (131.05–320.848)	210.945 ± 47.172 (124.627–322.845)	− 0.327 ± 7.096 (− 18.25–18.491)
fold_1	LV Cavity	0.815 ± 0.087 (0.661–0.968)	0.696 ± 0.123 (0.493–0.939)	1.572 ± 0.917 (0.159–3.519)	− 1.628 ± 5.718 (− 30.061–5.743)	47.973 ± 18.991 (11.869–102.306)	46.345 ± 17.664 (11.595–100.975)	− 2.125 ± 9.692 (− 29.383–18.52)
fold_1	LV Myocardium	0.874 ± 0.06 (0.734–0.965)	0.781 ± 0.092 (0.58–0.932)	1.527 ± 0.866 (0.273–3.567)	0.209 ± 12.412 (− 27.573–30.925)	280.281 ± 66.751 (177.419–484.622)	280.49 ± 63.689 (174.627–479.978)	0.472 ± 4.956 (− 9.846–14.25)
fold_1	RV	0.865 ± 0.07 (0.594–0.957)	0.769 ± 0.101 (0.423–0.917)	1.702 ± 1.032 (0.385–6.0)	− 0.652 ± 11.98 (− 29.931–29.048)	202.83 ± 43.794 (122.975–304.494)	202.178 ± 41.765 (121.363–312.566)	0.067 ± 5.954 (− 12.774–16.234)
fold_2	LV Cavity	0.833 ± 0.095 (0.528–0.973)	0.724 ± 0.129 (0.359–0.947)	1.433 ± 1.073 (0.206–5.247)	− 0.85 ± 5.047 (− 13.078–13.049)	53.764 ± 35.244 (11.869–231.013)	52.914 ± 35.392 (11.16–236.785)	− 1.164 ± 9.452 (− 17.504–18.551)
fold_2	LV Myocardium	0.883 ± 0.061 (0.676–0.963)	0.796 ± 0.094 (0.511–0.928)	1.385 ± 0.933 (0.273–4.131)	4.981 ± 15.773 (− 35.023–49.504)	282.916 ± 64.301 (160.504–480.007)	287.898 ± 67.382 (163.585–515.493)	1.793 ± 5.44 (− 9.931–18.462)
fold_2	RV	0.877 ± 0.057 (0.724–0.967)	0.785 ± 0.088 (0.568–0.935)	1.559 ± 0.923 (0.312–4.112)	1.086 ± 12.474 (− 36.354–23.855)	209.898 ± 47.477 (128.765–352.168)	210.984 ± 47.312 (117.38–359.748)	0.637 ± 5.884 (− 14.231–12.1)
fold_3	LV Cavity	0.832 ± 0.087 (0.55–0.961)	0.721 ± 0.122 (0.379–0.926)	1.497 ± 1.063 (0.19–5.076)	− 1.347 ± 4.387 (− 13.844–6.741)	53.906 ± 21.291 (11.869–112.316)	52.56 ± 20.088 (10.948–109.409)	− 1.987 ± 7.893 (− 18.693–19.067)
fold_3	LV Myocardium	0.882 ± 0.062 (0.641–0.962)	0.794 ± 0.094 (0.472–0.927)	1.459 ± 0.95 (0.243–5.052)	5.488 ± 16.645 (− 27.063–59.645)	298.16 ± 69.159 (158.421–493.649)	303.648 ± 73.697 (160.938–517.258)	1.764 ± 4.992 (− 8.007–16.334)
fold_3	RV	0.882 ± 0.056 (0.65–0.956)	0.794 ± 0.085 (0.482–0.915)	1.495 ± 0.876 (0.313–5.142)	2.449 ± 13.803 (− 27.544–33.764)	211.996 ± 45.636 (121.546–320.848)	214.444 ± 45.622 (124.323–318.621)	1.385 ± 6.681 (− 11.934–17.115)
fold_4	LV Cavity	0.829 ± 0.098 (0.525–0.971)	0.719 ± 0.135 (0.356–0.943)	1.484 ± 1.08 (0.135–4.784)	− 2.128 ± 8.437 (− 47.214–8.781)	56.33 ± 32.943 (11.869–231.013)	54.203 ± 32.511 (11.234–235.96)	− 2.381 ± 12.432 (− 64.398–15.929)
fold_4	LV Myocardium	0.881 ± 0.064 (0.643–0.969)	0.793 ± 0.097 (0.474–0.941)	1.446 ± 0.973 (0.233–5.423)	5.474 ± 14.943 (− 28.889–48.769)	288.728 ± 75.468 (162.544–533.243)	294.203 ± 75.501 (162.486–511.818)	2.002 ± 5.414 (− 9.3–19.178)
fold_4	RV	0.88 ± 0.062 (0.663–0.965)	0.791 ± 0.094 (0.496–0.933)	1.59 ± 1.212 (0.28–7.733)	4.278 ± 11.958 (− 24.13–30.018)	206.39 ± 49.248 (127.969–352.168)	210.668 ± 51.774 (128.75–358.056)	1.954 ± 6.001 (− 12.006–16.917)
Overall	LV Cavity	0.828 ± 0.091 (0.525–0.973)	0.716 ± 0.127 (0.356–0.947)	1.481 ± 1.014 (0.135–5.247)	− 1.368 ± 5.652 (− 47.214–13.049)	52.417 ± 26.341 (11.869–231.013)	51.05 ± 25.73 (10.948–236.785)	− 1.695 ± 9.466 (− 64.398–19.865)
Overall	LV Myocardium	0.88 ± 0.063 (0.619–0.969)	0.79 ± 0.096 (0.448–0.941)	1.46 ± 0.954 (0.233–5.646)	3.887 ± 14.748 (− 35.023–59.645)	286.891 ± 66.628 (158.421–533.243)	290.777 ± 67.733 (160.938–517.258)	1.473 ± 5.186 (− 9.931–19.178)
Overall	RV	0.876 ± 0.062 (0.594–0.967)	0.785 ± 0.093 (0.423–0.935)	1.629 ± 1.249 (0.28–13.942)	1.146 ± 13.048 (− 42.994–33.764)	208.718 ± 46.985 (121.546–352.168)	209.864 ± 47.014 (117.38–359.748)	0.741 ± 6.4 (− 18.25–18.491)

**Fig. 3:**
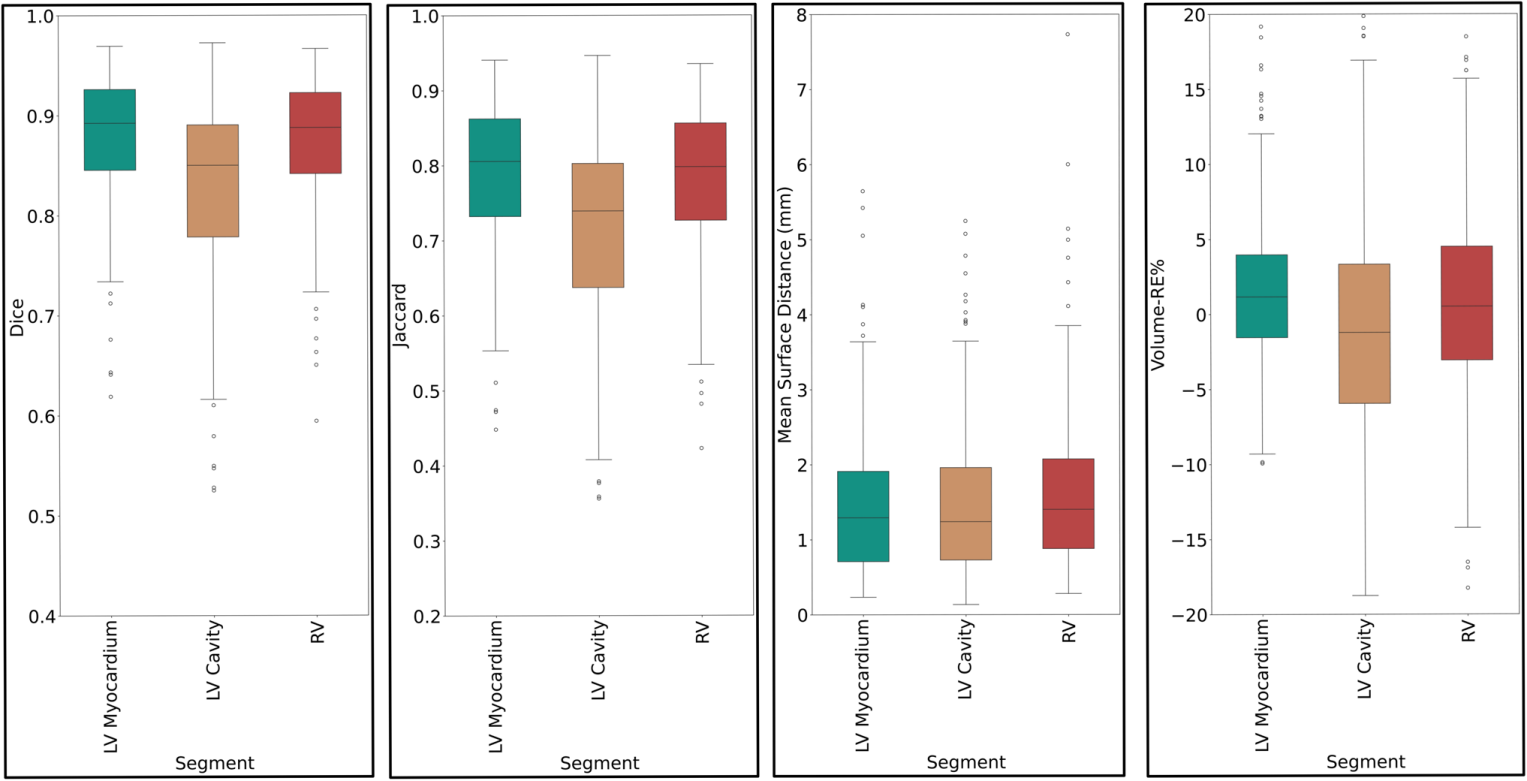
Box plots of task #2 fivefold internal validation evaluation metrics

Figure 3 shows the box plot of the different performance metrics, including the Dice coefficient, Jaccard, mean surface distance, and relative change in the volume of segmentations. The average Dice on the external data for task #2 was 0.884 ± 0.042, 0.837 ± 0.072, and 0.883 ± 0.083 for LM, LV, and RV, respectively. The average Dice on the external validation set was comparable since it reflects the ensembled inference of the five separate models.

**Fig. 4:**
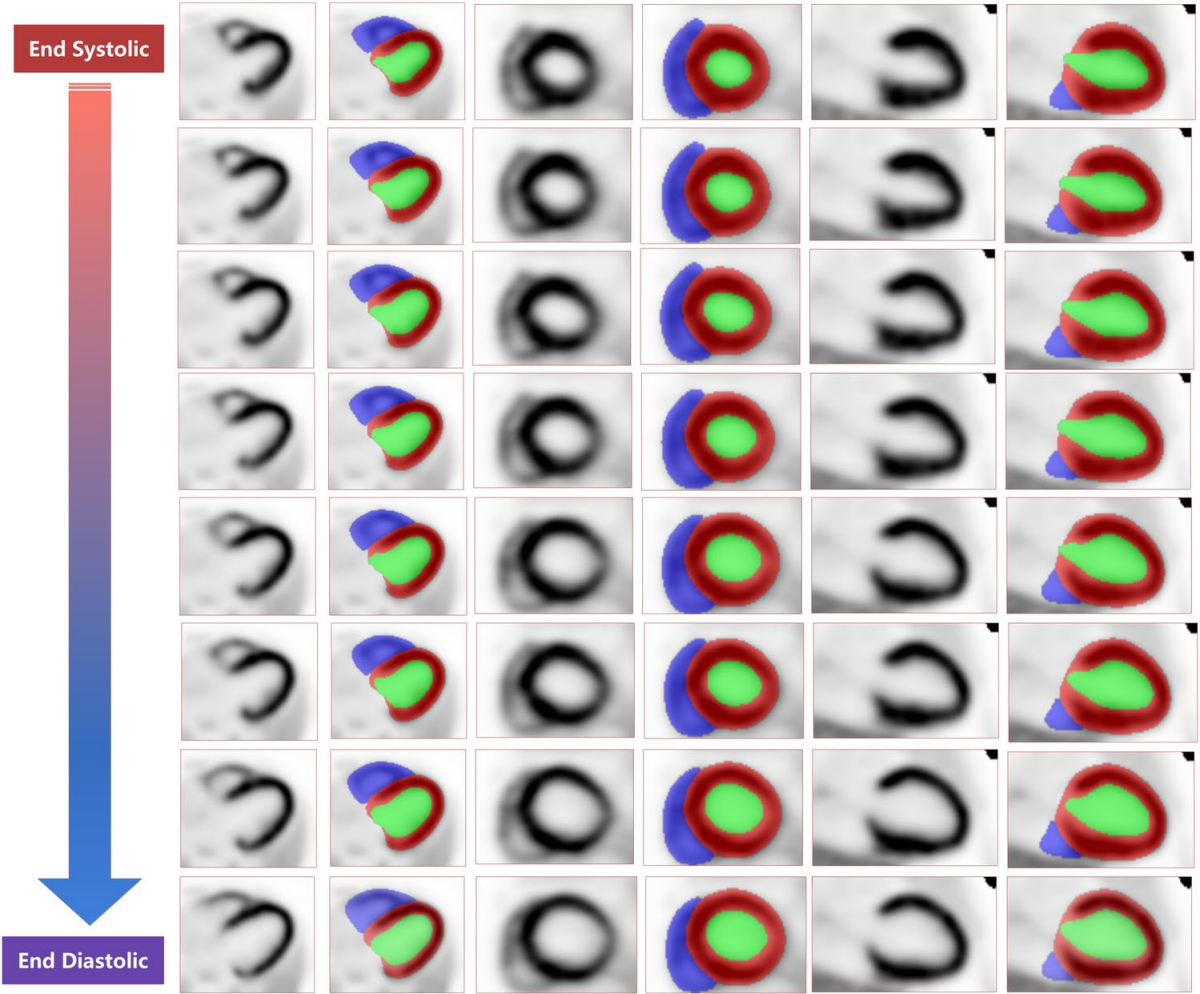
Segmentation visualization on a gated cardiac ^13^N-NH_3_ image. Blue, RV; green, LV cavity; red, LV myocardium. The top row is an end systolic, whereas the bottom row is an end diastolic image. The images were sorted from end systolic to end diastolic

Our model showed robust performance even on cases where there is non-homogenous uptake in the myocardium due to ischemia as shown in supplementary Fig. 1. Figure 4 shows examples of segmentations of a stress gated ^13^N-NH_3_PET image, sorted from end systole to end diastole. The model performance was acceptable with images reconstructed using parameters different from those used on the training data. An example of cavity volume changes is shown in supplementary Fig. 2. Kruskal–Wallis test showed significant differences for all metrics, except mean surface distance. Supplementary Fig. 3 summarizes the post-hoc statistical results.

**Fig. 5:**
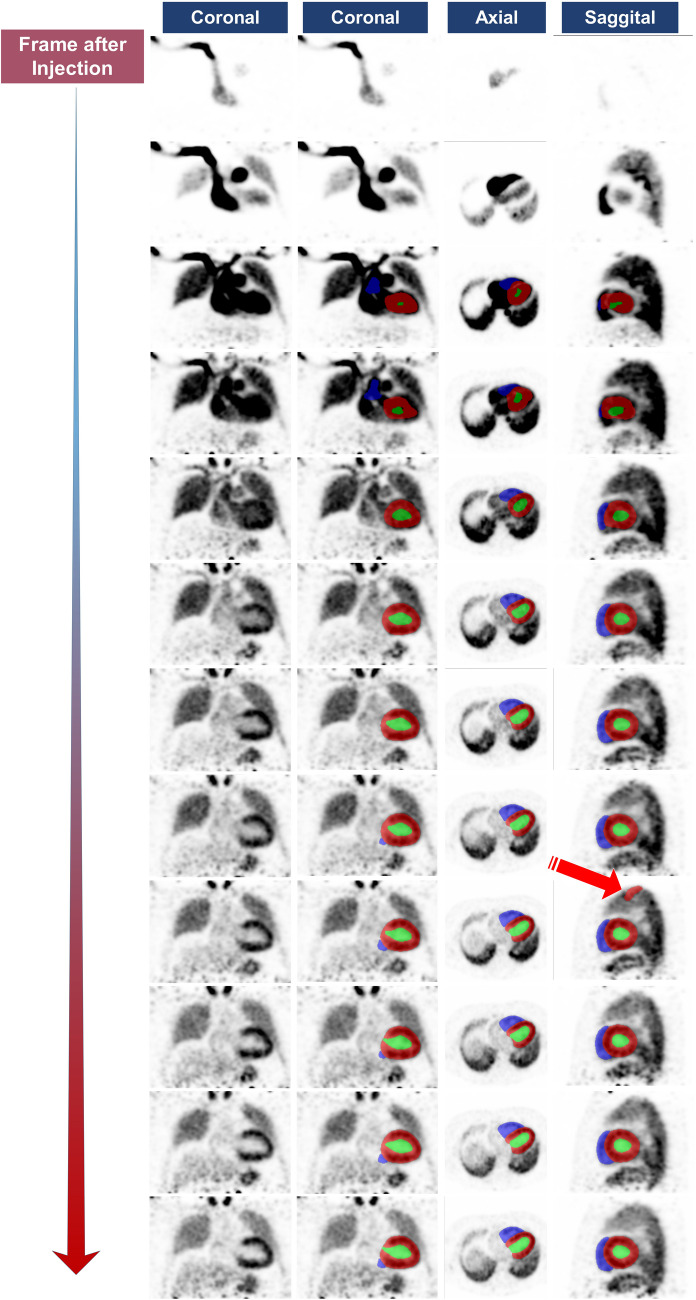
Example of dynamic first pass ^13^N-NH_3_ PET image segmented by our models from task #2. The red arrow shows a false positive segmented area outside the cardiac region as LM

We have tested our model on dynamic first-pass noisy images with 1 s per frame duration, and the results were acceptable and comparable with models output on gated single-phase images generated by CardioFreeze and the fivefold training summation images. A representative example of a noisy image is depicted in Fig. 5. In the first frames as there is no uptake in the heart, the model could not detect anything, but after few seconds, the model was able to segment the cardiac components. The arrow shows an example of area outside the cardiac region segmented as LV myocardium by our nnU-Net V2 model.

## Discussion

Robust and reliable segmentation of the heart is a requirement for PET/CT image quantification. However, available CT segmentation tools bear inherently a number of limitations in a clinical setting. Cardiac CTAC scans and in particular low-dose CTAC, commonly used in clinical practice, are usually acquired without contrast enhancement and, as such, have limited contrast for the delineation of cardiac cavities’ contours. Deep learning–based CT segmentation models may generate reasonable segmentations, but mismatch between the segmentation masks defined on CT images and PET myocardial uptake is very frequent [30]. The complicated movement involving changes in cardiac shape and position between CT and PET images limits the possibility of manual co-registration of PET image and CT segmentations. In this study, we first attempted to explain and understand the reasons behind the low performance achieved by DL algorithms in cardiac segmentation, then according to the obtained results, we developed a DL-based pipeline based on PET images to overcome this limitation.

There are multiple causes limiting the application of DL-based CT segmentation in hybrid cardiac PET/CT imaging; one reason may be possible errors in CT segmentation models on low-dose and ultra-low-dose CTAC images as reported by Tsanda et al. [31]. Another reason could be phase mismatch between PET and CT images resulting from cardiac and respiratory motion. Our evaluations and experiments showed that errors in DL-based low-dose CTAC segmentation and unvoluntary motion mismatch between PET and CT images contribute to the fact that the CT generated segmentations are not optimal in hybrid cardiovascular PET/CT imaging (Fig. 2), particularly when evaluating gated images where in the best case scenario in which there is no error in DL-based low-dose CT segmentations; the CT-generated segments are matched only with one of the gated PET images. According to our results, even when using a random image, nnU-Net reproduces a comparable shape with the actual image which emphasizes the importance of shape features in DL-based segmentation and makes it less reliable for unenhanced noisy CT images. In summary, the CT-generated cardiac masks are not reliable for three main reasons, namely, the mismatch between summation (static) PET and CT images, the poor accuracy of DL-based CT segmentation on noisy low-dose CT images, and the mismatch between cardiac gated PET and CT images.

Reliable and accurate CT-less cardiac PET segmentation offers significant clinical utility across diverse scenarios by delineation of cardiac components. Examples of possible applications include the following:

1. Quantification of LVEF and wall motion abnormalities using gated cardiac PET images. This approach eliminates misalignment errors inherent to CT-based segmentation in dynamic cardiac imaging.
2. Evaluation of myocardial blood flow and first pass imaging using dynamic cardiac PET with high temporal resolution. The proposed method demonstrates robust performance on noisy, low-count dynamic frames as shown in Fig. 5.
3. Cardiac imaging protocols use low- or ultra-low-dose CT for attenuation correction, where CT image quality is insufficient for reliable segmentation. A CT-independent methodology ensures accurate quantification without reliance on suboptimal CT images.
4. Scenarios where CT imaging is unavailable, e.g., in deep learning-based CT-less PET imaging. The method provides standalone PET-based segmentation without compromising accuracy.
5. Direct comparison of multiple tracers’ uptake within segmented cardiac regions. The model’s consistent performance across tracers facilitates tracer-agnostic quantitative analysis.
6. Evaluation of RV volume, motion, and function in conditions, such as pulmonary hypertension or congenital heart disease.
7. Radiomics and prognostic modeling by extraction of radiomic features from segmented PET images to enable risk stratification, such as predicting arrhythmias in cardiac sarcoidosis or outcomes in heart failure. Automated segmentation ensures reproducibility for high throughput radiomic pipelines.
8. Quantification of ischemic burden or infarct extent using reoriented PET images can be used for evaluation of perfusion pathologies, such as myocardial infarction and stress-induced ischemia. The segmentation supports defect localization and severity grading, critical for therapeutic decision-making.
9. Integration into automated pipelines for high-volume clinical or research applications in the framework of large-scale or automated clinical workflows.

We explored two different strategies to overcome PET/CT cardiac segmentation challenges, segmenting both the whole heart and its separate components. The whole cardiac segmentation model (task #1) showed superior performance compared to the cardiac components model (task #2), which enables post-processing based removal of false positive voxels segmented outside the cardiac region by task #2 model as suggested by Salimi et al. [10]. An example of outlier false positive voxel detected as LV myocardium is shown in Fig. 5 on dynamic noisy images, which was outside the whole cardiac segmentation and can be easily removed using task #1 model, making the proposed model less prone to outliers. In addition, task #1 segmentation may be useful in dynamic cardiac imaging quantification. We have tested our model on dynamic first-pass noisy images with 1 s per frame duration, and the results were acceptable and comparable with the models tested on the gated single phase summation images generated by CardioFreeze software (Siemens Healthineers) and the summation images used for training. This fact shows the robustness of our models against image noise, making it applicable to noisy dynamic images. Our method is capable of ejection fraction and wall motion quantification with excellent accuracy as the average volume relative error in all cardiac components’ segmentation was less than 2%. It should be emphasized that our model can generate gated volume changes, ejection fraction, and end diastolic and end systolic volume, as well as motion curves for both the right and left ventricle. An example of a volume curve is shown in supplementary Fig. 2. We included mean surface distance and volume relative difference as two metrics reflecting the real application of segmentation masks. As PET image cardiac wall information may be different in images with an ischemic area on the cardiac wall or absence of tracer uptake due to previous myocardial infarction (MI) as reported in [21], we included ischemia positive cases in our train and external data. Supplementary Fig. 1 shows an example of this kind of images with acceptable model performance. One other limitation of using PET images for the delineation of cardiac tissue could be the liver or any other extra cardiac activity overlapping the inferior wall that can affect the model’s accuracy as shown in supplementary Fig. 1, and Figs. 1 and 5. Our model was able to exclude these uptakes and delineate the cardiac tissue with excellent accuracy. Besides, using task #1, whole cardiac segmentation can overcome this issue in case of an outlier in external unseen set. Our results were consistent over three different tracers with no statistically significant difference among them. Even with the low number of ^82^Rb PET images included in training, the models’ performance on external images was comparable, which could be related to the similarity in cardiac uptake and the strength of nnU-Net models to produce reliable segmentations. In contrast to Zhang’s [24] and Entezarmahdi et al. [19] methods, our workflow achieved fully automated segmentation without any manual steps, accurately segmenting the three main heart components with comparable Dice coefficients. Unlike their approaches, which require cropped and reoriented short-axis cardiac images, and the use of other algorithms, such as QPS and QGS methods, our model processes raw PET data directly, thus eliminating the need for reorientation and cropping. This capability is particularly advantageous for the automated processing of large-scale datasets. We used the CT component of PET/CT images to generate reference segmentation masks for training our models, but the inference step requires only PET images and does not rely on CT images. An alternative approach consists in segmenting CT images and registering PET and CT images and transferring the CT segmentations to PET images. As described in Fig. 2, the misalignment cannot be tackled using rigid registration, and as such, deformable registration is mandatory. Moreover, this approach requires robust and reliable segmentation of CT images, which is not straightforward using common clinical PET/CT protocols involving the use of unenhanced low-dose CT scans as discussed in “Understanding Underperformance” in the Results. Although dedicated algorithms to segment noisy low-dose CT images have been developed [32], such approaches are not applicable to cardiac substructure segmentation. Indeed, image-to-image coregistration can tackle the misalignment between the summation PET and CT images. However, coregistration with gated PET images can be more challenging. These facts limit the application of image-to-image registration approaches and emphasize the importance and application of CT-less cardiac segmentation approaches.

The nnU-Net training pipeline is being used as a benchmark for comparison of different models, and the winners of most of segmentation challenges were using this pipeline. This pipeline selects the training hyperparameters of patch size, normalization, and voxel spacing by considering the training data and uses deep supervision and optimized UNET architecture to adopt the model to the considered clinical problem.

In comparison with the available literature for the same task, our results in terms of Dice score were superior to Entezarmahdi et al. [19] and lower than Zhang et al. [24]. However, it should be noted that the input to their models were the reoriented cropped cardiac images which require manual preprocessing, while our methodology is fully automated operating directly on 3D PET images. Our study concluded that CT segmentations are not reliable for cardiac PET quantification, primarily due to two factors: limitations of unenhanced, low-dose CT segmentations and misalignment between these segmentations and gated cardiac PET images. Future studies may focus on developing more robust CT-less segmentation algorithms using larger datasets and novel deep learning architectures. We have shared our trained models on GitHub. Performing transfer learning or fine-tuning them on local data could also be an interesting option.

This study inherently bears several limitations. The number of training data was relatively small, and we mixed three different tracers to demonstrate reliable robust performance across different tracers used in cardiovascular molecular imaging. We have shared the models publicly on our GitHub page and other groups and users may evaluate the models’ performance on their local dataset. All images included in this study were from three different Siemens PET/CT scanners. Further testing on images from other manufacturers is necessary to guarantee robustness and generalizability of our models.

## Conclusion

We developed a pipeline enabling to delineate the whole heart and cardiac components from PET images of three different cardiac tracers with an acceptable performance. The proposed pipeline could be a solution to overcome unreliable segmentations generated on CT images, which are frequent in the clinic, with an inference time of less than a minute for a gated cardiac study. The trained models and inference instructions are available on GitHub at https://github.com/YazdanSalimi/Organ-Segmentation.

## Supplementary Information

Below is the link to the electronic supplementary material.

Supplementary file1 (PDF 186 KB)

## Data Availability

The data used in this work is not available. The trained segmentation models and inference instructions would be Available on GitHub platform.

## Abbreviations

PET: Positron emission tomography
SPECT: Single-photon emission computed tomography
CT: Computed tomography
SUV: Standardized uptake value
DL: Deep learning
SD: Standard deviation
RV: Right ventricle
LV: Left ventricle
LM: Left myocardium
