# Supplementary material for: Deep Learning-Based CT-Less Cardiac Segmentation of PET Images: A Robust Methodology for Multi-Tracer Nuclear Cardiovascular Imaging
Source: J Imaging Inform Med. 2025 May 6;39(1):933–47. doi: 10.1007/s10278-025-01528-0 (PMC12921079; doi:10.1007/s10278-025-01528-0)
Supplement: Supplementary file 1 — Supplementary file1 (PDF 186 KB) [file 10278_2025_1528_MOESM1_ESM.pdf]

Supplementary material

Supplementary figure 1 shows the model output on a case with non-uniform uptake of tracer in the myocardium diagnosed as stress induced ischemia.

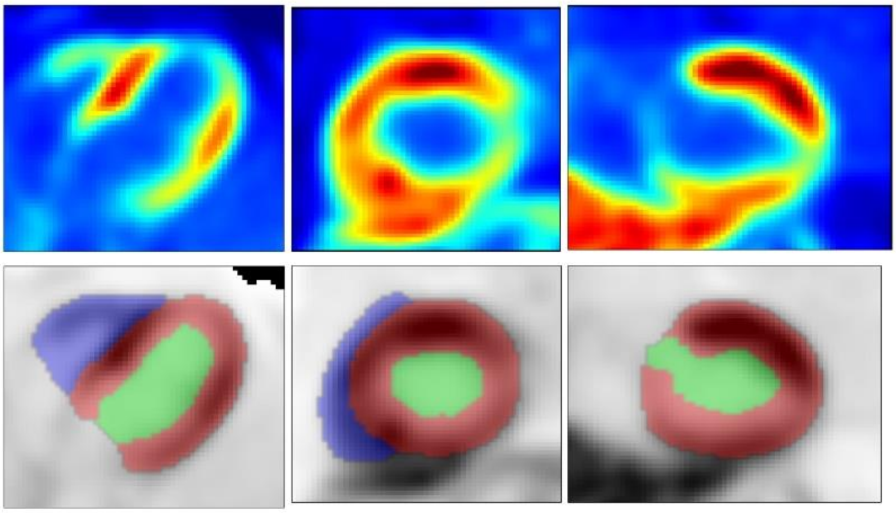

Supplementary Figure 1. Our model output on a case with cardiac ischemia.

Supplementary figure 2 shows the left and right ventricle volumes calculated on a gated image.

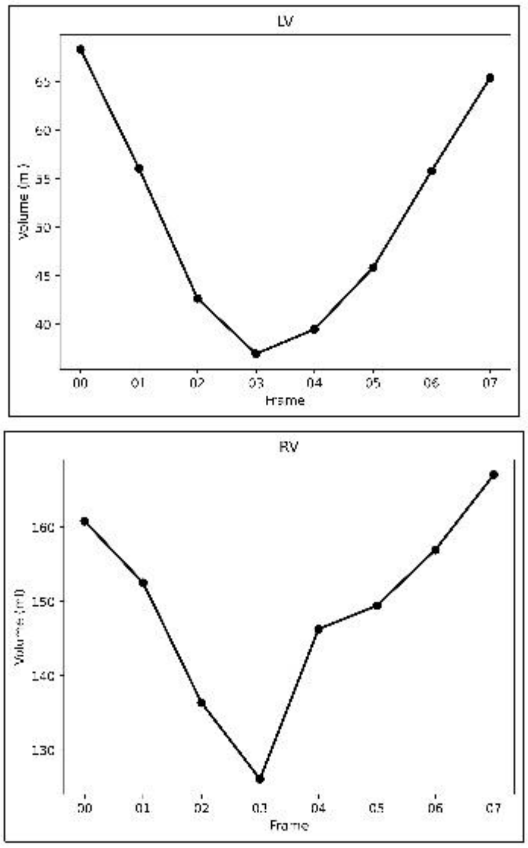

Supplementary Figure 2. Right and Left ventricle volume changes curve on a gated images showing the volume changes.

Supplementary figure 3 shows the results of post-hoc test and the statistical differences between the segmentation evaluation metrics for three sub cardiac structures delineated with our model. From 406 image and segmentation pairs evaluated visually, 308 of them were accepted without changes and the rest were corrected manually. The correction consisted in removing voxels segmented outside the target region.

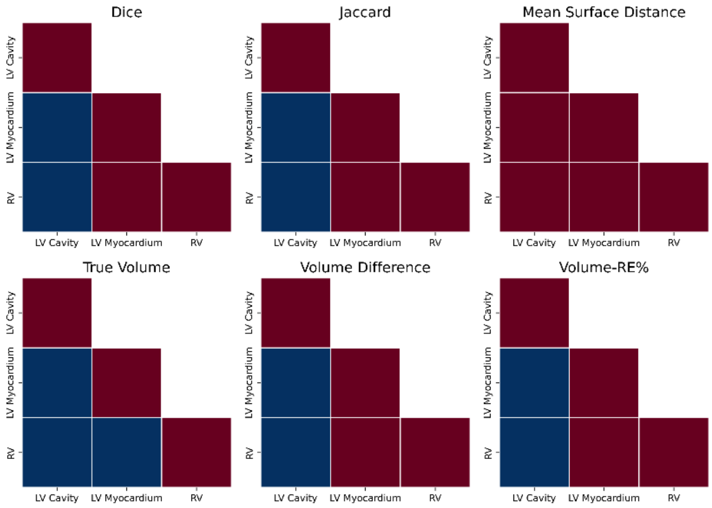

Supplementary Figure 3. Results of post-hoc test showing the differences among three segmentation masks separated by the segmentation evaluation metrics. Dark red means no statistical difference while dark blue means the presence of statistical difference between two groups.
